# Efficacy of Trimetazidine in the Prevention of Contrast-Induced Nephropathy in Patients Undergoing Contrast Coronary Intervention: A Systematic Review and Meta-Analysis (PRISMA)

**DOI:** 10.3390/jcm13072151

**Published:** 2024-04-08

**Authors:** Tiny Nair, Saumitra Ray, Jacob George, Arindam Pande

**Affiliations:** 1 Department of Cardiology, PRS Hospital, Thiruvananthapuram 695002, Kerala, India; 2 Invasive Cardiology, AMRI Hospital, Kolkata 700019, West Bengal, India; drsaumitra@yahoo.co.in; 3 Department of Nephrology, Trivandrum Medical College, Thiruvananthapuram 695011, Kerala, India; drjacobgeo@gmail.com; 4 Department of Cardiology, Medica Superspeciality Hospital, Kolkata 700099, West Bengal, India; drapande@gmail.com

**Keywords:** trimetazidine, CIN, cardiac intervention

## Abstract

**Objective**: The present systematic review assessed the efficacy of peri-procedurally administered trimetazidine in the prevention of contrast-induced nephropathy (CIN) in patients undergoing coronary interventions with contrast agents. **Methods**: We performed a systematic literature review of articles published in PubMed and Google Scholar by 7 December 2023 and included articles from the last 15 years that evaluated the efficacy of trimetazidine in preventing CIN in cardiac patients undergoing coronary intervention. **Results**: After title/abstract and full-text screening, this systematic review included 9 randomized controlled trials (N = 2158 patients) with two groups: Trimetazidine (60–70 mg/day 24 to 48 h before and up to 72 h after the procedure) with hydration and the control group with only hydration. A total of 234/2158 patients developed CIN (Incidence rate [IR], 10.8%) as per the CIN definition of the Contrast Media Safety Committee of the European Society of Urogenital Radiology. The incidence of CIN in the trimetazidine vs. control group was 6.4% (69/1083) vs. 15.4% (165/1075), and the odds ratio (95% CI) was 0.3753 (0.279–0.504). **Conclusions**: In conclusion, the trimetazidine group had a lower incidence of CIN. Trimetazidine offers a reno-protective effect and helps in reducing the CIN incidence in patients undergoing cardiac intervention. Peri-procedure administration of trimetazidine significantly decreases the risk of CIN in patients despite comorbidities.

## 1. Introduction

The use of contrast media in coronary interventions is almost ubiquitous and the quantity of contrast exposure increases with the complexity of coronary lesions. This use of contrast agents in interventional procedures such as coronary angiography (CAG), percutaneous coronary intervention (PCI), and percutaneous valve replacement procedures can lead to acute kidney injury (AKI), especially for patients with pre-existing renal disease or comorbidities such as hypertension, diabetes, or hyperlipidemia. This clinical condition of impairment of kidney function after administering contrast media is identified as contrast-induced nephropathy (CIN) [1].

CIN may affect 1.6% to 2.3% of those undergoing diagnostic interventions but is known to affect up to half of the high-risk patients undergoing coronary intervention [2]. It is an important cause of hospital-acquired kidney injury, especially in those with risk factors such as hypertension, diabetes mellitus, previous myocardial infarction, higher age, damaged left anterior descending artery, Killip class of 2 or higher, lower left ventricular ejection fraction (LVEF), reduced glomerular filtration rate, etc. [3]. CIN is associated with increased morbidity as well as mortality following cardiac procedures such as CAG or PCI. The occurrence of CIN post-contrast can lead to an increase in hospital stay and medical costs along with long-term kidney damage [4]. Therefore, it is important to prevent the development of CIN in patients undergoing complex coronary intervention.

The Contrast Media Safety Committee (CMSC) of the European Society of Urogenital Radiology (ESUR) has defined CIN as an increase in serum creatinine (SCr) by at least 25% or 44 μmol/L within 3 days after contrast media administration in the absence of an alternative etiology [5]. The guidelines recommended the use of hydration pre- and post-procedure to reduce the risk of CIN. The guidelines also mention the lack of definitive evidence of any particular pharmacological intervention. However, ongoing clinical research focuses on the efficacy and safety of different interventions for preventing CIN including hydration with sodium bicarbonate rather than normal saline and the use of drugs such as N-acetyl cysteine, high-dose statins, and trimetazidine [5,6,7]. 

The rationale of this systematic review is to evaluate the efficacy of one such intervention by trimetazidine in the prevention of CIN in patients undergoing CAG or PCI. Since its introduction in 1969, trimetazidine, a metabolic modulator, has demonstrated a cardioprotective effect in patients with angina, diabetes mellitus, and left ventricular dysfunction. It has demonstrated efficacy in revascularization procedures while maintaining a tolerable safety profile. Trimetazidine is an attractive choice for both patients and clinicians given that it does not interfere with the heart rate, arterial pressure, and common comorbidities [8]. 

Trimetazidine has been credited with preserving phosphocreatine and ATP intracellular levels, reducing cell acidosis, calcium overload, and free radical–induced injury caused by ischemia. The pathogenesis of CIN includes oxygen-free radical release and ischemic injury to kidney tissue. It is speculated that trimetazidine has a protective effect on the kidney in ischemic injury leading to CIN. By enhancing mitochondrial activity in the kidney, trimetazidine reduces the incidence of CIN. Specifically, it reduces oxygen-free radical release thus reducing the toxicity of contrast agents to renal tubular epithelial cells [9].

A previous systematic review has assessed the published evidence on the efficacy of trimetazidine in the prevention of CIN in patients undergoing CAG or PCI. In this recent systematic review and meta-analysis [2], trimetazidine was associated with a reduced risk of CIN; however, the study was finalized in October 2020. Two major clinical trials evaluating the same research question have been published recently [4,9]. Both these trials have added to the available evidence based on their large sample sizes of 760 patients with diabetes [4] and 310 patients with renal insufficiency [9]. Thus, we decided to conduct the present systematic review and synthesize the data provided by new relevant publications. We believe that a new systematic review is needed in view of possible publications of new data with larger sample sizes that may provide more robust results.

The objective of this systematic review is to focus on the clinical trial evidence on the efficacy of trimetazidine in the prevention of CIN in patients undergoing CAG or PCI. Since the guidelines are often modified periodically, we have included clinical trials in the past 15 years to evaluate the impact of the administration of trimetazidine on the incidence of CIN in patients undergoing cardiac interventions with contrast.

## 2. Materials and Methods

**Figure 1:**
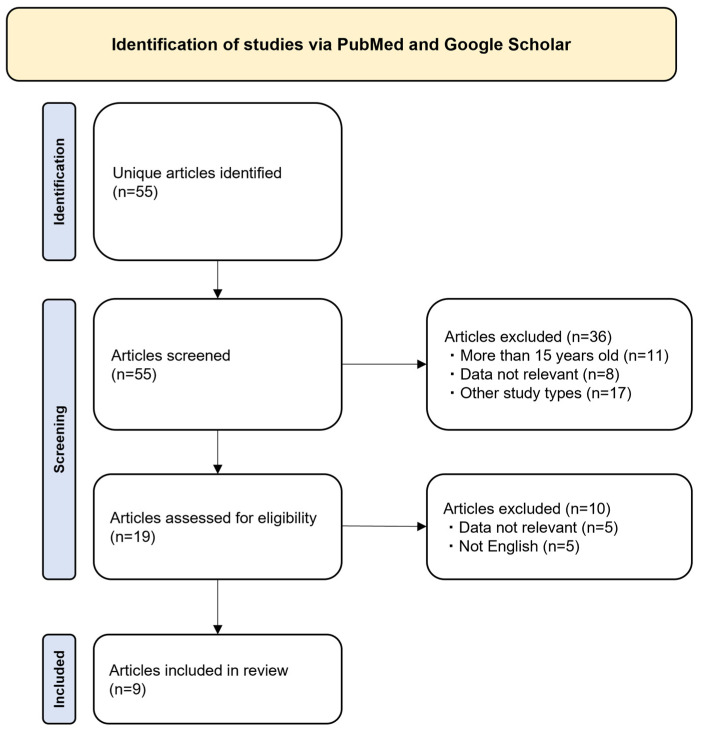
Preferred reporting items for systematic reviews and meta-analyses (PRISMA)-style flowchart of study selection and review.

This systematic review is reported as per the Preferred Reporting Items for Systematic Reviews and Meta-Analyses (PRISMA) guidelines (Figure 1).

### 2.1. Search Strategy

A systematic literature search was conducted to obtain trials assessing the effect of trimetazidine on the incidence of CIN in patients undergoing cardiac interventions such as CAG or PCI. A search string capturing different aspects of the research question was developed to retrieve articles from MEDLINE PubMed and Google Scholar. The search string was used to focus on two facets: trimetazidine and nephropathy, using different keywords. The different tenets of the search strategy are described below, keeping the PICOTS guidelines recommended by PRISMA:

Patient (P)—Patients undergoing Cardiac Intervention (CAG, PCI, or coronary artery bypass graft [CABG]);
Interventions (I)—Administration of trimetazidine; peri-procedure;
Comparator (C)—Not restrictive; included placebo, only hydration, or active comparator;
Outcomes (O)—Incidence of CIN (an increase in SCr of ≥25% or ≥44 μmol/L within 3 days after administration of contrast media, in the absence of an alternative etiology [5];
Time Frame (T)—15 years;
and Study type (S)—Only clinical trials.
The literature search was performed in March 2023 and updated on 7 December 2023.

A search string capturing different aspects of the research question was developed to retrieve articles from MEDLINE PubMed. We used filters for humans and for articles published in the English language only. Systematic literature reviews, meta-analyses, narrative reviews, non-randomized or observational studies, case series, case trials, editorials, and commentaries were excluded. Also, an additional search was performed with Google Scholar. The keywords in the search string were (trimetazidine) AND (nephropathy OR kidney OR renal OR creatinine). To keep the search broad, we did not add the term “contrast” in it. We also hand-searched the bibliographies of relevant articles to identify additional studies.

### 2.2. Study Selection

Two authors (TN and SR) independently evaluated the titles and abstracts of the identified published articles for relevance to the aim of this systematic review and published in the past 15 years. Subsequently, full-text publications were evaluated in a similar manner. The articles were screened based on the inclusion and exclusion criteria mentioned above. Any discrepancies in the decision were discussed until a consensus was reached.

### 2.3. Data Extraction

The relevant data on study characteristics, efficacy, and safety were extracted. Wherever possible, data were summarized by patient subgroups and by risk (e.g., those with diabetes or renal insufficiency). The data on efficacy were summarized based on the CIN incidence within 48–72 h of contrast media use. The data on change in the levels of creatinine clearance rate (CrCl), blood urea nitrogen (BUN), SCr, and cystatin-C (Cys-C) were analyzed, where available. The safety endpoints were also recorded.

### 2.4. Statistical Analysis

The incidence rate (%) of CIN in trimetazidine and control groups using the extracted data was calculated. Furthermore, the overall bivariate odds ratio (OR) and 95% confidence interval (CI) with a *p*-value of 0.05 using trimetazidine as an independent (explanatory) variable and incident CIN as a dependent (outcome) variable were calculated. The online tool available on vassarstats.net was used for the statistical computation of OR, CI, and *p*-value. ReviewManager (RevMan Web Version: 7.4.0) was used for creating the Forest plot. The Cochrane Risk of Bias Tool for Randomized Controlled Trials (RCTs) was used for the assessment of bias for each included article. The heterogeneity of included articles was assessed visually by inspecting the Forest plot (eyeball test) and statistically using the *I*^2^ statistic, with a random-effects model. 

## 3. Results

### 3.1. Study Selection

A total of 55 possibly relevant articles from PubMed and Google Scholar (Figure 1) were identified. At the title/abstract stage, we excluded 36 studies: the studies that were published before 1 March 2008 (n = 11), studies that were not relevant (n = 8; no CIN data, n = 6 and no trimetazidine data, n = 2) and other study types such as meta-analysis, systematic reviews, or narrative reviews (n = 17), after which 19 articles were identified as relevant. During the evaluation of the full texts of these 19 articles, 10 articles were excluded: 5 articles did not have relevant CIN data and 5 were not in English. After these exclusions, 9 most relevant articles were included for detailed synthesis and review as per PRISMA guidelines (Figure 1).

### 3.2. Study Characteristics

The study characteristics of the 9 included articles [4,6,9,10,11,12,13,14,15] are provided in Table 1. All 9 trials were controlled trials. Of these, 8 were identified as RCTs, while the details of randomization could not be ascertained for 1 trial [14]. Only hydration was given to the control group while hydration with trimetazidine was administered to the trimetazidine group. The trimetazidine-to-control ratio was approximately 1:1 in all the trials, with total sample sizes across trials ranging from 100 to 760 patients. Of the 9 included articles, 5 were from China, 2 from Egypt, and 1 each from Iran and Bangladesh. In 8 of 9 trials, the trimetazidine group received a minimum dose of 60 mg trimetazidine daily pre- and post-procedure.

The trials included a total of 2158 adults of both genders. Most trials included patients with coronary heart disease who underwent elective cardiac catheterization CAG or PCI. All trials included patients with comorbidities. One trial was conducted in the general population [10], 3 trials were specifically conducted in patients with diabetes [4,13,15] and the remaining 5 trials were conducted in patients with renal insufficiency or chronic kidney disease [6,9,11,12,14]. Generally, the trials excluded patients with acute myocardial infarction receiving emergency PCI, receiving trimetazidine 7 days prior to the procedure, severe cardiac insufficiency (LVEF < 30%), cardiogenic shock, and heart failure, hypersensitivity to trimetazidine, and severe liver damage/autoimmune diseases malignant tumor/infectious diseases. Some trials have specifically excluded patients with diabetes [10] while some trials have a diagnosis of diabetes as the inclusion criteria [4].

Type of contrast agent used: Use of iso-osmolar or low-osmolar iodine agents was reported in 7 of the 9 trials. No trial reported the use of any high-osmolar contrast agent. Iodixanol is an iso-osmolar iodine agent while iopromide and iopamidol are low-osmolar iodine agents. One trial [9] used both iopromide and iodixanol as the contrast agent. Three trials used only iodixanol as the contrast [4,11,15] while two other trials used only iopromide as the contrast agent [6,13]. One trial used iopamidol [10] and 2 trials did not report the details of the contrast agent used [12,14].

### 3.3. Patient Characteristics

Table 2 displays the baseline characteristics of patients included in the trials. Mean age varied from 56 years to 77 years, 65% of patients were males while only 35% were females. Approximately 64% of patients were diagnosed with diabetes while 55% of patients had hypertension as a comorbidity at the time of participation in the study. The mean LVEF was >50% in 5 studies and was not reported in 4 studies. The baseline serum creatinine levels were comparable across the two groups.

### 3.4. Efficacy Results

#### 3.4.1. Overall Efficacy

**Figure 2:**
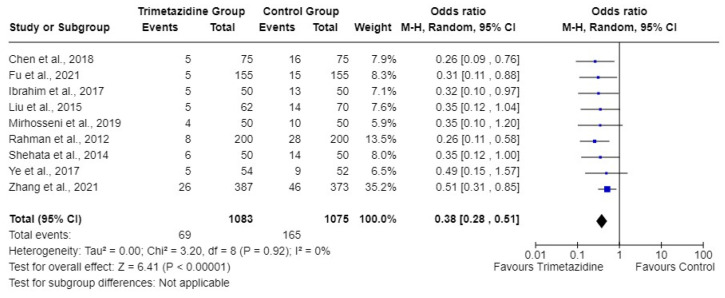
Forest plot of nine articles demonstrating the effectiveness of trimetazidine in the prevention of contrast-induced nephropathy [4,6,9,10,11,12,13,14,15]. M-H, Mantel-Haenszel.

Of 2158 patients, 1083 received trimetazidine along with hydration (trimetazidine group) while 1075 received only hydration peri-procedure (control group). Combined CIN incidence was 6.4% in the trimetazidine group vs. 15.4% in the control group. The overall (calculated) bivariate OR for CIN was 0.3753 (95% CI: 0.2794–0.504) for the trimetazidine group vs. the control group (*p*-value < 0.0001) (Figure 2). The addition of trimetazidine to standard hydration had reduced the incidence of CIN in these patients.

Table 3 summarizes the efficacy results. Three trials [4,6,9] provided OR; another 5 trials [10,11,12,14,15] presented the incidence rate of CIN in patients treated with trimetazidine vs. control groups. The online computation tool available on vassarstats.net was used to calculate the OR, CI, and the corresponding *p*-values.

#### 3.4.2. Subgroup Analyses

**Age:** Six of 9 trials [4,6,9,12,14,15] had a mean age of >60 years. A significant reno-protective effect of trimetazidine was seen in all these trials except Mirhosseni et al., 2019 (N = 100) and Ye et al., (N = 106) [12,15], which showed a trend towards protective OR, though non-significant.

**Gender:** Of the total 2158 patients, 65% patients were males while only 35% were females. However, gender-wise results were not discernible from the publications.

**Renal insufficiency:** Seven of 9 trials had renal insufficiency/CKD as inclusion criteria. Two of these trials with sample size ≥150 demonstrated the risk of CIN in the trimetazidine group to be approximately 70% lower than that in the control group [6,9]. The other 5 trials with smaller sample sizes of <150 patients showed a reno-protective trend [11,12,13,14,15].

**Diabetes:** One study included 760 patients with diabetes undergoing PCI. The incidence of CIN in the control group was more than the trimetazidine group (12.3% vs. 6.7%) demonstrating the protective effect of trimetazidine in the diabetic subgroup. The OR for CIN in the trimetazidine vs. placebo group was 0.294 (95% CI: 0.094–0.920; *p*-value = 0.035) [4].

**LVEF:** Patients with low LVEF (30–40%) were excluded from the trials; therefore, the role of trimetazidine in patients with low cardiac output cannot be commented on.

Overall, trimetazidine with hydration consistently showed a reno-protective effect across all subgroups.

### 3.5. Safety Results

All trials recorded some side effects or major adverse cardiac events, such as acute heart failure, malignant ventricular arrhythmia, emergency redo PCI, acute hemodialysis, cerebrovascular events, bleeding, or CABG after the primary procedure. Also, all-cause mortality occurring during the index hospitalization and within 14 to 30 days post-procedure was recorded. Across the trials, the occurrence of adverse events in both the control group and the trimetazidine group were generally comparable. However, one article [11] mentioned a significantly lower incidence of 12-month adverse events post-CAG in the trimetazidine group vs. the control group (9.6% vs. 22.8%; *p*-value = 0.043); the Kaplan–Meier survival curves of adverse events demonstrated a significantly lower incidence of adverse events in trimetazidine vs. control group (log-rank *p*-value = 0.035). Ye et al. also recorded the major adverse cardiovascular events (MACE) in 106 patients and observed that the incidence of MACE in the trimetazidine group was significantly lower than that in the control group (7.41% vs. 18.51%) and (*p*-value < 0.05) [15].

### 3.6. Assessment of Included Articles

#### 3.6.1. Risk of Bias

**Figure 3:**
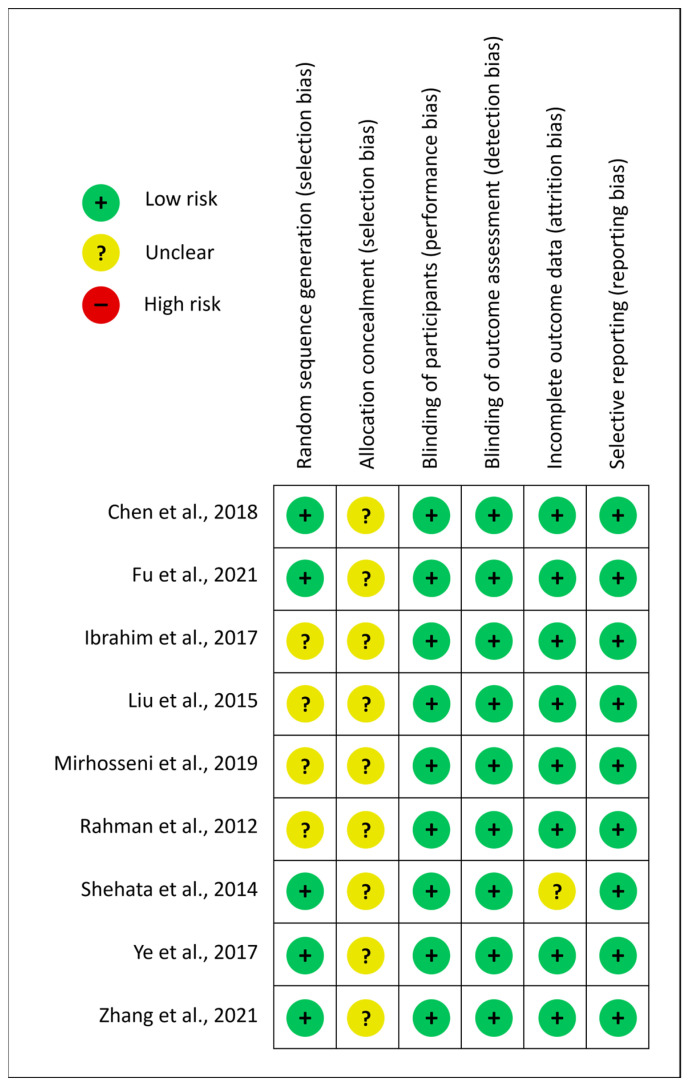
Cochrane risk of bias assessment [4,6,9,10,11,12,13,14,15].

Each article was assessed using the Cochrane bias assessment tool. Overall, the included articles were found to be of fair quality, with ≤2/6 biases being assessed as of unclear risk and ≥4/6 biases demonstrating low risk (Figure 3).

#### 3.6.2. Heterogeneity

Visual interpretation of 95% CI for ORs of included articles, using the eyeball test revealed low heterogeneity among the included articles (Figure 2). Statistical heterogeneity was low, with *I*^2^ of 0%.

## 4. Discussion

In this systematic review, the efficacy of trimetazidine in preventing CIN was evaluated. CIN is defined as an increase in SCr of ≥25% or ≥44 μmol/L within 3 days after administration of contrast media, in the absence of an alternative etiology [5]. The incidence of CIN is highly dependent on renal function prior to contrast media administration and additional risk factors, of which diabetes mellitus is the most important one [16]. Research also suggests that the use of low osmotic and iso-osmotic contrast media is better for the prevention of CIN [9]. In the present review, 7 out of 9 trials had used low/iso-osmotic contrasts, viz., iopromide/iodixanol (details of contrast used were not reported for 2 trials) (Table 1). With the use of low or iso-osmolar iodine contrasts, the development of CIN did not vary materially across studies based on the type of contrast media. Since the exact contrast media used, whether low-osmolar or iso-osmolar, in particular patient populations in these articles was not clear, an evaluation of the role of iso-osmolar vs. low-osmolar contrast media in the development of CIN was not possible.

Our results provide robust evidence adding to the previous systematic review and meta-analysis by Behzadi et al. focusing on patients with renal insufficiency [2]. In contrast to this previous meta-analysis [2] that included a total of 1611 patients from 11 articles, our present analysis included robust data from 2158 patients from 9 articles published in the English language only within the last 15 years. Three non-English articles and 1 old article from the previous meta-analysis [2] did not meet our inclusion criteria, while we included 2 recently published articles [4,9] that were not a part of the previous meta-analysis [2]. Despite these differences, the results of both meta-analyses are generally consistent demonstrating that add-on trimetazidine reduces the risk of CIN in patients undergoing coronary interventions with contrast agents. Trimetazidine, with the chemical name of 1-(2,3,4-trimethoxybenzyl)-piperazine, was historically developed as an anti-myocardial ischemia drug to improve myocardial energy metabolism in 1969 [4]. Experimental trials have reported that during cellular ischemia trimetazidine retains the intracellular concentration of ATP and inhibits the extracellular leakage of potassium [12].

Three distinct renal mechanisms, viz, medullary ischemia, formation of reactive oxygen species, and direct tubular cell toxicity are postulated to be involved in the pathophysiology of CIN. The exact contribution of each of these mechanisms towards the development of CIN in the individual patient remains unclear [17]. However, based on the assumption that reactive oxygen radicals and renal medullar ischemia may be involved in the pathogenesis of CIN, it could be assumed that trimetazidine may be useful in the prevention of CIN being an anti-ischemic agent with antioxidant properties [17].

In the 9 included trials a reno-protective effect of trimetazidine when administered in the peri-procedure period (24–48 h prior and 48–72 h after) was plausible, with lower incidence of CIN in the trimetazidine group vs. control group. The combined CIN incidence was 6.4% in the trimetazidine group vs. 15.4% in the control group. However, the bivariate ORs were not significant and crossed unity for 4 trials with smaller sample sizes of <150 patients [11,12,13]. Nevertheless, when the data from all 9 trials were used to calculate the overall bivariate OR, the results demonstrated that the risk of CIN was significantly less (62.5% less) compared to the placebo group (95% CI: 0.2794–0.504), (*p*-value < 0.0001). The reno-protective effect of trimetazidine was consistently evident across subgroups of patients based on baseline characteristics of age, gender, renal insufficiency, and diabetes. The reno-protective effect was seen in the trials with a mean age of >60 years, suggesting trimetazidine could be useful in the elderly age group. Diabetes is one of the major risk factors for the development of CIN in patients undergoing coronary procedures. One article included 760 patients with diabetes undergoing PCI and demonstrated a 71.6% reduced risk of CIN in the trimetazidine group compared with the control group after adjusting for all relevant variables [4]. In our review, 7 of 9 trials had renal insufficiency/CKD as inclusion criteria. Two of these trials with sample size ≥150, demonstrated that the risk of CIN in the trimetazidine group was approximately 75% lower than that in the control group [6,9]. The calculated bivariate ORs showed a protective trend but were not significant and crossed unity for 3 trials with smaller sample sizes of <150 patients [11,12,13]. We could not assess efficacy by gender as gender-wise results were unavailable from the publications. However, the trials included 65% males indicating that males are prone to develop a cardiac condition necessitating CAG or PCI. Patients with low LVEF (30–40%) were excluded from the trials; therefore, the role of trimetazidine in patients with low cardiac output cannot be commented on. A small clinical study in India suggests a reno-protective effect of trimetazidine in renal insufficiency patients. Out of the 140 patients with baseline serum creatinine of more than 1.5 mg/dL demonstrated that at the end of 7 days, the serum creatinine levels in the trimetazidine group were significantly lower than the non-trimetazidine group. A drop of creatinine was found to be 12.6% and 8.8%, respectively [18]. Overall, trimetazidine with hydration consistently showed a reno-protective effect across subgroups. This suggests that trimetazidine could be administered in the peri-procedure period in patients undergoing CAG or PCI to reduce the incidence of CIN.

CIN is an important implication of the contrast used in patients undergoing cardiac intervention with contrast. Developing CIN can increase the hospital stay, and healthcare costs. Even though current guidelines recommend the use of only hydration to prevent CIN, an addition of drugs like trimetazidine which also have a safe profile can provide added protection to these patients with comorbidities such as diabetes and renal insufficiency. Therefore, a more detailed and bigger sample size RCT is recommended to evaluate the effectiveness of the addition of trimetazidine to standard hydration in patients undergoing cardiac intervention with contrast.

Calculation of bias and heterogeneity: Overall, the included articles demonstrated low heterogeneity both visually and statistically (*I*^2^ = 0%). The 95% CI for all ORs overlapped substantially with the combined OR, demonstrating low heterogeneity as per the eyeball/visual test. Also, all included trials showed low clinical heterogeneity as the interventions (trimetazidine + hydration vs. hydration only) were fairly uniform across the trials, the outcome measurements (CIN, defined by CMSC as an increase in serum creatinine by at least 25% or 44 μmol/L within 3 days after contrast media administration, in absence of an alternative etiology) were uniform, and also 8/9 articles included patients with renal insufficiency and/or diabetes (except one article [10] which included general population). All the included articles were clinical trials, and of fair quality with low risk of bias as assessed by the Cochrane Risk of Bias Tool, thus demonstrating low methodological heterogeneity.

### Limitations

The results of this review should be assessed in light of potential limitations. The current evidence is based mainly on trials with a small sample of patients. Eight out of 9 trials had renal insufficiency and/or diabetes as the inclusion criteria, so patients without comorbidities are underrepresented in this meta-analysis. Furthermore, the efficacy benefit demonstrated is relatively small and should be weighed against the risks and costs associated with the use of trimetazidine. A separate cost-effectiveness analysis is warranted to answer this question.

The dose of trimetazidine used (20 mg TDS/35 mg BD) was as per the anti-angina dose of trimetazidine. The effect of loading dose with a single high dose can be evaluated in a separate trial to optimize the trimetazidine dose to prevent CIN.

Nevertheless, synthesis of the available data suggests that trimetazidine demonstrates a potential benefit in preventing CIN in patients undergoing CAG or PCI and shows a clear reno-protective effect in a clinical trial setting.

## 5. Conclusions

Our systematic review and meta-analysis have synthesized the available evidence on the efficacy of trimetazidine in CIN prevention and demonstrated the significant reno-protective effect of trimetazidine when administered in the peri-procedure period in patients undergoing contrast cardiac interventions along with standard hydration. The pooled results showed that the risk for CIN was reduced by >60% (OR: 0.3753 [95% CI: 0.279–0.504) for the trimetazidine group compared to the control group. Efficacy is observed across all patient groups, including those with renal dysfunction.

## Figures and Tables

**Table 1:** Study characteristics.

Sr No	Author	Study Design	Geography	Trimetazidine Dose	Population Characteristics, Specific Inclusion Criteria	Procedure Performed	Type of Contrast	Sample Size	
								Control	Trimetazidine
1	Chen et al., 2018 [6]	RCT, Double Blind paralleled	China	20 mg, TDS for 48 h pre and 72 h post-procedure	Renal Insufficiency	CAG/PCI	Iopromide	75	75
2	Fu et al., 2021 [9]	RCT	China	20 mg, TDS for 24 h pre and 72 h post procedure	Renal Insufficiency	PCI	Iopromide /Iodixanol	155	155
3	Ibrahim et al., 2017 [14]	RCT	Egypt	35 mg, BD for 48 h pre and 24 h post procedure	Renal Insufficiency	CAG	NA	50	50
4	Liu et al., 2015 [11]	RCT	China	20 mg, TDS for 48 h pre and 24 h post procedure	Renal Insufficiency	CAG/PCI	Iodixanol	70	62
5	Mirhosseni et al., 2019 [12]	RCT	Iran	35 mg, BD for 48 h pre and 24 h post procedure	Renal Insufficiency	CAG	NA	50	50
6	Rahman et al., 2012 [10]	RCT	Bangladesh	35 mg, BD for 96 h starting at a 48 h pre procedure	General	CAG	Iopamidol	200	200
7	Shehata et al., 2014 [13]	RCT, Double blind	Egypt	35 mg, BD for 72 h starting at a 48 h pre procedure	Mild CKD + Diabetes	PCI	Iopromide	50	50
8	Ye et al., 2017 [15]	RCT	China	20 mg, TDS for 48 h pre and 24 h post procedure	Diabetes + Renal insufficiency	CAG/PCI	Iodixanol	52	54
9	Zhang et al., 2021 [4]	RCT	China	35 mg, BD for 24 h pre and 72 h post procedure	Diabetes	PCI	Iodixanol	373	387

BD, twice daily; CAG, coronary angiography; CKD, chronic kidney disease; h, hours; NA, data not available; PCI, percutaneous coronary intervention; RCT, randomized controlled trial; TDS, thrice daily.

**Table 2:** Patient characteristics.

Sr No	Author	Sample Size, N	Mean Age, Years	Male, n (%)	Mean LVEF, %	Mean Serum Creatinine (Trimetazidine vs. Control), μmol/L	Diabetes, n (%)	Hypertension, n (%)
1	Chen et al., 2018 [6]	150	62	83 (55)	55	NA	65 (43)	67 (45)
2	Fu et al., 2021 [9]	310	77	152 (49)	NA	101.27 vs. 100.94	141 (45)	177 (57)
3	Ibrahim et al., 2017 [14]	100	64	57 (57)	52	137.94 vs. 138.82	57 (57)	64 (64)
4	Liu et al., 2015 [11]	132	59	75 (57)	NA	107.74 vs. 103.38	80 (61)	72 (55)
5	Mirhosseni et al., 2019 [12]	100	66	44 (44)	51	112.27 vs. 114.04	62 (62)	63 (63)
6	Rahman et al., 2012 [10]	400	56	336 (84)	NA	122.88 vs. 123.76	NA	316 (79)
7	Shehata et al., 2014 [13]	100	59	68 (68)	54	176.80 vs. 176.80	100 (100)	48 (48)
8	Ye et al., 2017 [15]	106	64	63 (59)	NA	NA	106 (100)	75 (71)
9	Zhang et al., 2021 [4]	760	66	527 (69)	60	^†^	760 (100)	300 (39)
	**Overall**	**2158**	**-**	**1405 (65)**	**-**	**-**	**1371 (64)**	**1182 (55)**

^†^ 62.1 vs. 63.5; 97.8 vs. 100.0; and 117.8 vs. 114.0 μmol/L for those belonging to low-, moderate-, and high-risk groups, respectively; LVEF, left ventricular ejection fraction; NA, data not available.

**Table 3:** Efficacy results.

Sr No	Author	Trimetazidine Group				Control Group				Bivariate OR (Calculated)		Multivariate OR (From Publication)	
		Yes	No	N	% Incidence	Yes	No	N	% Incidence	Bivariate OR (95% CI)	*p*-Value	Bivariate OR (95% CI)	*p*-Value
1	Chen et al., 2018 [6]	5	70	75	6.7	16	59	75	21.3	0.2634 (0.091–0.762)	0.017	0.252 (0.082–0.774)	0.016
2	Fu et al., 2021 [9]	5	150	155	3.2	15	140	155	9.7	0.3111 (0.1102–0.8785)	0.035	0.274 (0.089–0.847)	0.025
3	Ibrahim et al., 2017 [14]	5	45	50	10	13	37	50	26	0.3162 (0.1032–0.9686)	0.0373	NA	NA
4	Liu et al., 2015 [11]	5	57	62	8.1	14	56	70	20	0.3509 (0.1185–1.0391)	0.080	NA	NA
5	Mirhosseni et al., 2019 [12]	4	46	50	8.0	10	40	50	20	0.3478 (0.1012–1.1954)	0.148	NA	NA
6	Rahman et al., 2012 [10]	8	192	200	4.0	28	172	200	14	0.256 (0.1136–0.5767)	0.0007	NA	NA
7	Shehata et al., 2014 [13]	6	44	50	12.0	14	36	50	28	0.3506 (0.1223–1.005)	0.078	NA	NA
8	Ye et al., 2017 [15]	5	49	54	9.3	9	43	52	16.7	0.4875 (0.1517–1.5668)	0.2608	NA	NA
9	Zhang et al., 2021 [4]	26	361	387	6.7	46	327	373	12.3	0.512 (0.3094–0.8472)	0.009	0.294 (0.094–0.920)	0.035
	**Overall**	**69**	**1014**	**1083**	**6.4**	**165**	**910**	**1075**	**15.4**	**0.3753** **(0.2794–0.504)**	**<0.0001**	**-**	**-**

CI, confidence interval; NA, data not available; OR, odds ratio.

## Data Availability

The data presented in this study are available in reference material in the article.
